# Fecal Glucocorticoid Metabolite Responses of Brown Kiwi (*Apteryx mantelli*) to Ambassador Program Participation and Translocation: Implications for Captive Management and Welfare

**DOI:** 10.3390/ani15081156

**Published:** 2025-04-17

**Authors:** Kathleen Brader, Natalia A. Prado, Janine L. Brown, Mary Kearney, Nicole Boisseau, Lisa Ware, Kristina M. Delaski, Wesley Bailey

**Affiliations:** 1 Department of Animal Care Sciences, Smithsonian National Zoo and Conservation Biology Institute, Washington, DC 20008, USA; 2 Biology Department, College of Arts and Science, Adelphi University, Garden City, NY 11530, USA; 3 Endocrinology Research Laboratory, Center for Species Survival, Smithsonian National Zoo and Conservation Biology Institute, Front Royal, VA 22630, USA; 4 Wildlife Health Sciences, Conservation Medical Unit, Smithsonian National Zoo and Conservation Biology Institute, Front Royal, VA 22630, USA; 5 Department of Animal Programs, Smithsonian National Zoo and Conservation Biology Institute, Front Royal, VA 22630, USA

**Keywords:** welfare, corticosterone, zoo, education, physiology

## Abstract

The brown kiwi (*Apteryx mantelli*) is a flightless, nocturnal bird native to New Zealand and is classified as “At Risk” due to predation from domestic dogs and ferrets. In the U.S., brown kiwi have been managed under the AZA Animal Population Management and Studbook Program since 2006, with the Smithsonian National Zoological Park (NZP) and Conservation Biology Institute (SCBI) maintaining the species since 1968. However, because they are nocturnal, kiwi are not good exhibit animals and often are difficult for zoo visitors to observe during the day. To address this, the NZP launched a “Meet-A-Kiwi” ambassador program in 1989 to engage the public and raise awareness. The program ran successfully for 28 years until 2017, when renovations closed the Bird House at the zoo in Washington, DC, and the birds were moved to the Front Royal, VA campus. Over time, concerns about the suitability of kiwi as ambassador birds arose as they are nocturnal and do not imprint on people. This study assessed the effects of outreach events, housing, and relocation on adrenal activity in five kiwi (four males, one female) by measuring fecal glucocorticoid metabolite (fGCM) concentrations, a physiological stress indicator, from March to October 2016. Two males participated in outreach (ambassadors), while two males and one female did not (controls). The results showed no significant differences in fGCM concentrations between ambassador and control birds, suggesting that outreach did not cause undue stress. However, individual factors (age, sex, hatching type, and display status) were associated with differences in fGCM concentrations, highlighting the need for personalized management. Further longitudinal studies are needed to explore the physiological responses of kiwi to captive conditions.

## Simple Summary

This study examined how public outreach events, housing, and relocation affected adrenal activity in brown kiwi (*Apteryx mantelli*), flightless birds from New Zealand of conservation concern. Because kiwi are nocturnal and difficult to see during the day, the Smithsonian National Zoological Park (NZP) initiated a “Meet-A-Kiwi” program in 1989 to educate visitors. However, over time, concerns arose about whether kiwi were suited for this role. To assess the physiological response of brown kiwi at NZP to visitors, fecal glucocorticoid (GC) metabolites (fGCMs) were analyzed from March to October 2016. Two male kiwi participated in outreach events (ambassadors), while two males and one female did not (controls). The results showed that participating in outreach events did not significantly affect fGCM concentrations in ambassadors compared to control birds, suggesting that outreach did not cause undue stress in the birds. However, factors like age, sex, and hatching status (wild vs. captive) did, suggesting that kiwi have highly individualized physiological responses to their captive environment that warrant further research to better understand GC dynamics in this species.

## 1. Introduction

The brown kiwi (*Apteryx mantelli*) is a flightless, nocturnal bird endemic to New Zealand and belongs to the ratite group, which includes the emu, rhea, cassowary, and ostrich [1]. In recent decades, brown kiwi populations have declined significantly [1]. Projections suggest that, without sustained conservation efforts, the species may face extinction in the wild within the next few decades [1,2,3]. This decline is primarily attributed to predation by introduced species, notably domestic dogs and ferrets (*Mustela furo*), which have reduced the life expectancy of brown kiwi by approximately one-third [4]. Despite the challenges faced by wild populations, ex situ conservation efforts have shown promising results [1,2,3]. While not commonly exhibited outside of New Zealand, the captive population of brown kiwi in the United States has demonstrated gradual but consistent growth since 2006, coinciding with the species’ inclusion in the Association of Zoos and Aquariums (AZA) animal population management program [5], which includes an international studbook coordinated by W. Bailey. Currently, the ex-situ population consists of 58 birds across 17 zoological programs.

Kiwi have many traits unusual for an avian species: they are flightless, have solid bones, nares at the end of their bill, an unusually powerful sense of smell [6], two functioning ovaries [7], and specialized feathers that appear to act as whiskers [8]. Compared to other avian species, kiwi eggs are enormous, occupying up to 20% of the female’s body [9,10], and they incubate at a relatively low temperature; keepers delay feeding for several days post-hatching, unlike other birds that are fed immediately (W. Bailey, unpublished [11]). Their nocturnal nature makes them difficult to manage in traditional zoo settings [12], and there can be tension between the educational and conservation objectives of zoological programs and the entertainment expectations of visitors [13]. Kiwi are not easily acquired because they are a protected species and national symbol of New Zealand; so, the government strictly regulates exports to ensure their conservation. Kiwi also are notoriously difficult to breed in captivity [14]. They have a slow reproductive rate, with females typically laying only one or two eggs yearly [9]. The incubation period is 2–3 times longer than comparatively sized birds, lasting 75 to 90 days [9]. These factors make it challenging to increase captive populations. Finally, although most brown kiwi in the U.S. today are zoo-born and hand-reared, they do not imprint on humans [15], reducing their effectiveness as display or ambassador animals. The combination of legal restrictions, breeding challenges, specialized care requirements, limited sources, and ethical considerations make it exceptionally difficult for zoos to acquire kiwi for display, especially outside of New Zealand (K. Brader, unpublished [16]).

The Smithsonian National Zoological Park (NZP) in Washington, DC, has maintained a kiwi population since 1968 and, in 1975, was the first facility outside New Zealand to hatch a kiwi successfully [5]. However, the next kiwi would not hatch at NZP until 2006 [5]. In the intervening years, NZP acquired a 1-year-old male kiwi from the San Diego Zoo and initiated innovative outreach and education programs titled “Meet-A-Kiwi” and “Meet-and-Greet”. Ambassador animals are individuals of a species that are trained or habituated to interact with the public for educational purposes. The experience is facilitated by a staff member that pairs animal demonstrations with facts about the animal’s biology, life history, or conservation status. The kiwi ambassador program operated continuously for over 27 years until the Bird House closed for renovations in 2017, prompting the relocation of all birds to the Smithsonian Conservation Biology Institute (SCBI) in Front Royal, VA. At the NZP, the weekly “Meet-A-Kiwi” program was designed to provide visitors with an up-close encounter with a kiwi while educating them about this iconic species through interactions with knowledgeable keepers. During VIP “Meet-and-Greet” events, guests could interact closely with the kiwi, including opportunities to touch and hand-feed the bird. At the SCBI in Front Royal, VA, the ambassador kiwi was featured in VIP tours and at an annual 2-day open house event.

Concerns have been raised about the potential stress imposed on birds used in conservation programs. Stressful circumstances in vertebrates trigger the hypothalamic–pituitary–adrenal (HPA) axis, leading to increased glucocorticoid (GC) secretion from the adrenal glands [17]. In birds, this process involves corticosterone secretion stimulated by adrenocorticotropic and corticotropin-releasing hormones [18]. GCs play a crucial role in energy regulation, affecting several metabolic processes [19], including redirecting energy from non-essential functions like reproduction and digestion toward survival and stress recovery [20,21,22]. While these short-term responses are adaptive, prolonged stress can lead to detrimental effects, including suppressed reproduction and immune function, muscle wasting, neuronal death, and reduced growth [19,22,23,24]. For instance, Silverin [25] demonstrated that elevated plasma corticosterone levels during the nestling period significantly reduced reproductive success in adult pied flycatchers (*Ficedula hypoleuca*). In a study of great tits (*Parus major*), behavioral changes occurred when the birds were exposed to either a stuffed Tengmalm’s owl (a predator) or a stuffed passerine bird (a non-predator) for 60 min; however, elevated corticosterone concentrations were linked to nest and territory abandonment only in the presence of the predator [26]. Additional studies have reported increased corticosterone responses to handling in both wild and captive birds, suggesting that capture and handling may be perceived as a form of predation [18,26,27,28].

Blood sampling is common for avian corticosterone monitoring; however, this invasive method of collection can in itself induce a stress response [18,29,30]. Non-invasive hormone monitoring through the collection of excrement provides baseline hormone concentrations without interfering with the bird’s behavior [29], and produces hormone metabolite concentrations that reflect circulating concentrations [29,30,31]. Thus, fecal GC metabolite (fGCM) analyses offer a method of examining physiological responses over time to environmental changes that could help inform zoo programs [29,30,32].

The objective of this study was to examine relationships between fGCM concentrations and factors such as environmental noise, outreach events, and translocation in brown kiwi housed at NZP and SCBI. We hypothesized that (1) kiwi used in the ambassador program were habituated and so would not exhibit differences in mean fGCM concentrations compared to control birds (i.e., non-ambassador birds), (2) birds housed at NZP and on public display with more exposure to the public would exhibit higher fGCM concentrations than birds housed at SCBI with limited human contact, (3) older birds would have higher fGCM concentrations compared to younger birds, (4) birds that were captive-hatched would exhibit lower fGCM responses than those that were wild-hatched, (5) birds relocated from NZP to SCBI would demonstrate a short-term increased adrenal GC response, and finally (6) anthropogenic noise disturbances above normal background would be associated with higher fGCM concentrations.

## 2. Materials and Methods

### 2.1. Study Animals

Animal and study information is presented in Table 1. The study included five kiwi, designated as NZP#1–3 and SCBI#1–2, with one active ambassador bird at each facility. The NZP cohort consisted of three males. NZP#1, the ambassador bird, was hatched at NZP in 2012 and hand-reared. He was introduced to the demonstration box at 3 months of age and had been engaging in public encounters regularly since just over 1 year of age. NZP#2, a control bird, was wild-hatched in 1984 and arrived at NZP in 1991. While he was living alone during the study, he had previously been paired and served as a successful breeder for the brown kiwi management program. NZP#3, also a control bird, was captive-hatched at NZP in 1975, making him the first kiwi hatching outside New Zealand. He was hand-reared and housed on display at the Bird House at NZP since 1977. The SCBI cohort included two kiwi. The ambassador bird (SCBI#1) arrived at NZP in 1972 and served as an occasional ambassador at NZP before the arrival of NZP#1. SCBI#1 was transferred to SCBI in 2007 and has been the facility’s ambassador since 2010. The female control bird, SCBI#2, was captive-hatched in 2013 at SCBI, housed singly, and had never been paired or participated in outreach events. This study was exempt from Animal Care and Use Committee (ACUC) approval at NZP as it involved opportunistic, noninvasive sample collection conducted during routine keeper duties without direct animal contact or baiting.

### 2.2. Acclimatization Process for the Ambassador Kiwi Program

Kiwi chicks under 1 year of age were introduced to the program based on positive responses to hand-feeding, with a consistent keeper assigned for familiarity. The viewing box was prepared with earthworms or mealworms to create positive associations, and the exposure time was gradually increased from 5 to 10 min. The box was located in a demonstration room that allowed public observation without direct contact. After occasional program interruptions exceeding 2 months occurred early on and ambassador kiwi required 2–4 sessions to readjust to the routine, a behavior maintenance routine was implemented in which birds were placed in the viewing box (i.e., mock demonstrations) at least once a week to ensure continued acclimatization and readiness for demonstrations. A total of 5 birds have been used as ambassadors over the course of the program (K. Brader, unpublished [16]). The two ambassador kiwi at NZP and SCBI had been ambassadors for 4 and 6 years, respectively. Neither were used for breeding during the study.

### 2.3. Ambassador Outreach Events

During the study period, NZP#1 participated in 52 “Meet-A-Kiwi” events and 9 “Meet-and-Greet” events between 29 April and 30 July. The 1 h “Meet-A-Kiwi” events took place on Mondays, Wednesdays, and Fridays at 1100 h while keepers gave educational talks. NZP#1 was placed in a plexiglass demo box measuring 61 cm × 48 cm × 61 cm (Supplementary Photos S1 and S2). The floor had 7–10 cm of soil mix (peat moss, sterilite, and shredded mulch), which was kept moist. Earthworms and sometimes mealworms were provided as both a reward and motivator for the kiwi and to showcase its unique bill. Demonstration events for ambassador bird SNZP#1 ranged from 0 to 4 events per week; occasionally, both event types occurred on the same day. There was only one week when NZP#1 did not have any events during the study period. There were four ‘Meet-and-Greet’ events during the study period that were encounters with smaller groups of people of all ages who were allowed to touch and feed the kiwi (Supplementary Photo S3). ‘Meet-and-Greet’ group sizes ranged from 2 to 10 people, whereas ‘Meet-A-Kiwi’ audiences ranged from 8 to 100 people. Noise levels were collected at the end of each day during the study period (Table 2).

SCBI#1 participated in 45 1-h demonstrations, including one weekend at the Conservation Discovery Day event. For private tours, the ambassador kiwi would be picked up and held for 1–3 min by its keeper, depending on the kiwi’s perceived comfort, and then returned to its enclosure. At SCBI, large auditorium events were held annually during a pre-planned open house at SCBI, wherein the ambassador was in his demo box, covered by a sheet, backstage during the presentation, and brought to the front of the stage at the end for an introductory talk, after which guests were allowed to take turns coming up to see the bird up close (Supplementary Photos S1 and S2). Guests were asked to be quiet and not use flash photography. Demonstrations for SCBI#1 ranged from 1 to 3 events per week and never more than one per day. There was only one week with five events; this occurred during the Conservation Discovery Day weekend in early October 2016.

### 2.4. Gut Transit Time

The New Zealand Department of Conservation estimates a gut transit time of approximately 3 h for clinical assessments of brown kiwi ([33], p. 44). An opportunistic veterinary clinical case involving a young kiwi allowed us to confirm the estimated gut transit time through a barium sulfate series. Oral barium sulfate administration was conducted in a male kiwi (four months old) that presented with inappetence and a heavy load of coccidia. A barium sulfate series was conducted on 3 September 2016 to rule out gastrointestinal obstruction. Barium sulfate (7 mL, 10 mL/kg, Vet-Paque, JorVet, Loveland, CO, USA) was administered via gavage, and the bird was placed in a small, dark enclosure for imaging. Initial radiographs were taken every 15–20 min, with progressively increasing intervals. Barium sulfate was first observed pooling in the cloaca approximately 95 min after administration (Supplementary Image S1). Although the exact time of barium sulfate appearance in the feces was not recorded, it is likely that excretion began around this time. By 305 min post-administration, the barium sulfate was well distributed throughout the digestive tract (Supplementary Image S2). The final image was obtained at 23 h post-administration (Supplementary Image S3), showing that residual barium sulfate was still present but less dense. Fecal samples submitted on the same day series to Antech Diagnostics (Lake Success, NY, USA) revealed that no parasites were present. The observed transit time in the kiwi (~1.5 to 23 h) aligns with the New Zealand Department of Conservation recommendation of 3 h, as well as gut transit times in other ratites [34] such as the emu (*Dromaius novaehollandiae*) (5.5 h) [35,36] and cassowary (*Casuarius casuarius*) (3.3 to 26.9 h) [37].

### 2.5. Fecal Sample Collection

Fecal sample collection occurred from March to August 2016 for NZP birds and April to July 2016 for SCBI birds (Table 1). Sample collection for ambassador bird SCBI#1 continued into October 2016 to include an annual outreach event he participated in (Table 1). Opportunistic sample collection was resumed for NZP birds on 8 May 2017 due to translocation of two of birds (NZP#2 and NZP#3) to SCBI on 18 May 2017. Translocation samples were collected very other day, once a day, for 4–6 weeks for all NZP birds (Table 1).

Keepers aimed to collect feces five times a week for ambassador birds and three times a week for control birds (Table 1 and Table 3). Enclosures were checked daily, and the freshest-looking fecal samples were collected at 0600–0800 h for AM samples for all birds and at 1500 h for PM samples from ambassador birds only to investigate if a response in fGCMs would be observed after ambassador events (Table 3). PM sampling occurred at least 3 h after ambassador events to account for the gut transit time based on the results of the barium sulfate series described above. Whenever possible, keepers also collected fecal samples deposited in the demo box during ambassador events (i.e., DEMO samples) (Table 3). Unlike other avian species, ratite urine is stored and excreted separately from feces [38,39], and so urates are not typically mixed with deposited fecal material; thus, only fecal metabolites were measured in this study. The water content of fresh fecal samples was normalized by being freeze-dried (i.e., lyophilized) prior to hormone extraction. All collected fecal samples were placed into a small plastic bag labeled with the bird accession number, date and time of collection, and whether it was during a demonstration, and then stored frozen (−20 °C) until the hormone analysis at SCBI.

### 2.6. Fecal Hormone Metabolite Extraction

Samples were lyophilized, crushed with a mallet, and sifted with a fine mesh sieve to remove non-fecal debris. An aliquot (0.2 g +/− 0.01 g) was weighed into a glass tube (16 × 100 mm), followed by the addition of 5 mL of 80% ethanol. Tubes were vortexed briefly, placed on a multi-pulse shaker for 30 min, centrifuged at 1300× *g* for 20 min, and the supernatant decanted. Samples were extracted twice and the supernatants combined and dried. Once dry, 1 mL of 100% methanol was added to the glass tubes and sonicated briefly. Samples were dried a final time, reconstituted in 1 mL of assay buffer (Cat. No. X065, Arbor Assays, Ann Arbor, MI, USA), and frozen at −20 °C prior to the hormone analysis. To test the extraction efficiency, 100 µL of 3H corticosterone (~9600 dpm) was added to each sample prior to extraction. Following extraction, 50 µL of extract was combined with 3 mL of scintillation fluid and thoroughly shaken to homogenize the sample and measured with a suspension scintillation counter (Beckman Coulter LS6500, Fullerton, CA, USA). The mean extraction efficiency was 82% (range 64–99%).

### 2.7. Hormone Analyses

Concentrations of fGCMs were measured using a double-antibody enzyme immunoassay (EIA) that employed a polyclonal corticosterone antibody (CJM006, C.J. Munro, University of California, Davis, CA, USA) and horseradish peroxidase-labeled corticosterone as the tracer [40]. The CJM006 EIA has been previously validated for a variety of avian species, including blue-throated (*Ara glaucogularis*) and Spix’s (*Cyanopsitta spixii*) macaws, black vultures (*Coragyps atratus*), rockhopper penguins (*Eudyptes chrysocome*) [40], northern spotted owls (*Strix occidentalis caurina*) [41], and kea (*Nestor notabilis*) [42]. To validate the in house corticosterone enzyme immunoassay (EIA) for brown kiwi, parallelism and matrix interference assessments were conducted using linear regression [40]. Parallelism was demonstrated between serial dilutions of pooled samples with the standard curve for male (y = 0.748x + 4.901, R^2^ = 0.998, *p* < 0.0001) and female (y = 0.711x + 6.997, R^2^ = 0.998, *p* < 0.0001) birds in the study [42,43]. The recovery of the standard hormone added to pooled samples before the analysis averaged 83% (range: 69–99%), with no matrix interference (y = 0.993x + 7.185, R^2^ = 0.99, *p* < 0.0001) [42,43]. Additionally, individual profiles, the response to translocation, and demographic differences were assessed to confirm that the assay detected biologically relevant changes in fGCM concentrations.

The EIA was performed in 96-well microtiter plates pre-coated with anti-rabbit IgG. Dried fecal extracts were reconstituted in assay buffer (0.1 M phosphate-buffered saline containing 0.1% bovine serum albumin, pH 7.0) and diluted 1:80 before analysis. Standards (50 μL; range 0.078–20 ng/mL) or samples (50 μL) were added to wells in duplicate, followed by corticosterone-horseradish peroxidase (25 μL; 1:25,000) and antiserum (25 μL; 1:60,000), and incubated at room temperature for 1 h. After washing five times, 100 µL of substrate (TMB Peroxidase, Product # TMBHK-1000, Moss, Inc., Pasadena, MD, USA) was added to each well. The absorbance was measured at 405 nm using a microplate reader (Filtermax F5, Molecular Devices, Sunnyvale, CA, USA) after an incubation for 30–45 min at room temperature. The data are reported as ng/g dry feces. Intra- and inter-assay coefficients of variation were <10%. Assay sensitivity was 0.078 ng/mL or 0.45 ng/g dry weight, and no sample in this study fell below the detectable range of the assay.

## 3. Statistical Analyses

For each bird, summary measures were derived from the raw fGCM data using the R studio (v.4.1.2) package hormLong [44]. The following summary measures were obtained for each bird: mean, median, standard error of the mean (SEM), percent coefficient of variation (CV), minimum value (Min), maximum value (Max), baseline, base mean, and peak mean (Table 3). All statistical analyses were conducted using IBM SPSS software (IBM Corp. Released 2022. IBM SPSS Statistics for Windows, Version 29.0. Armonk, NY, USA: IBM Corp). For all tests described below, the dependent variable was the fGCM concentration. It is specified for each test whether summary measures (see Table 3), average weekly fGCM concentrations, or all data points were used. The independent variables (sample type, bird type, noise, event rate, total number of events, location, age, hatching status, or year of collection) changed depending on the test run. The data are expressed as means ± SEMs.

A univariate analysis of variance was conducted to examine the effect of the AM sample type on summary measures for all birds. The AM sample type was selected as a unifying criterion because control birds only had this sample type (Table 1), and it allowed us to control for GC differences due to possible circadian rhythm differences. There were no outliers, as assessed by an inspection of a boxplot. Normality was assessed using the Shapiro–Wilk normality test for each cell of the design (*p* > 0.05). All residuals were normally distributed.

A two-way ANOVA was conducted to examine the effects of all sample types (AM, PM, DEMO) and bird type (ambassador or control) on summary measures. A residual analysis was performed to test for the assumptions of the two-way ANOVA. Outliers were assessed by an inspection of a boxplot; normality was assessed using the Shapiro–Wilk normality test for each cell of the design. There were no outliers, and the residuals were normally distributed (*p* > 0.05). All pairwise comparisons were run, 95% confidence intervals reported, and *p*-values are Bonferroni-adjusted.

A one-way repeated measures ANOVA was conducted to examine the effect of the sample type (AM, PM, or demo) on summary measures (mean, median, percent CV, baseline, base mean, and peak mean) across ambassador birds NZP#1 and SCBI#1 only. There were no outliers, and the data were normally distributed by a visual assessment of a Q-Q plot of the mean data point. Epsilon (ε) was calculated for each variable according to Greenhouse and Geisser [45] and was used to correct the one-way repeated measures ANOVA. Post hoc analyses were run with a Bonferroni adjustment for each test.

Due to the small sample size and repeated measures, a Friedman test was run on all data to determine if there were differences in fGCM concentrations across multiple time points (AM vs. PM) within ambassador birds NZP#1 and SCBI#1. Pairwise comparisons were performed with the Bonferroni correction for multiple comparisons. Based on these results, linear regression was run to understand the effect of the time of day on all fGCM concentrations within ambassador birds. To assess linearity, a scatterplot of fGCM concentrations against the time of day was plotted. A visual inspection of the scatterplot indicated no linear relationship (R^2^ = 0.006) between the variables. There was homoscedasticity and normality of the residuals.

One-way ANOVA was conducted to determine if average weekly fGCM values differed with the number of events per week for ambassador birds NZP#1 and SCBI#1. Rates were calculated as the number of events over 7 days per week. There were no outliers, as assessed by a boxplot. The data were normally distributed for each group, as assessed by the Shapiro–Wilk test (*p* > 0.05). There was homogeneity of variances, as assessed by Levene’s test of homogeneity of variances (*p* = 0.200). The data are presented as means ± standard deviations.

Linear regression was run to further understand the effect of the total number of events on average weekly fGCM values per week within ambassador birds NZP#1 and SCBI#1. To assess linearity, a scatterplot of fGCM concentrations against the event rate was plotted. A visual inspection of the scatterplot did not indicate a linear relationship (R^2^ = 0.170) between the variables. There was homoscedasticity and normality of the residuals. The fixed variable was the number of events each week of the study period (range: 0–5) and the response variable was the average fGCM concentration. The test was run both with and without the outlier of one value for 0 events for NZP#1.

Linear regression was run to understand the effect of the group size (range: 2–100) on all fGCM data for ambassador NZP#1. To assess linearity, a scatterplot of fGCM concentrations against the group size was plotted. A visual inspection of the scatterplot indicated a slightly linear relationship (R^2^ = 0.080) between the variables. There was homoscedasticity and normality of the residuals. One outlier was present (group size of 100 people); the test was run twice, with and without the outlier present.

Due to the inability to normalize fGCM data and small sample sizes, non-parametric tests were used as follows: Mann–Whitney U tests compared fGCM concentrations across facilities (NZP vs. SCBI), exhibit status (on vs. off display), and hatching status (wild- vs. captive-hatched). Kruskal–Wallis H tests evaluated differences in fGCM concentrations across age groups and translocation effects on NZP birds. Pairwise comparisons for age groups were conducted using Dunn’s [46] procedure with the Bonferroni correction. fGCM distributions were visually assessed for similarity using boxplots. Finally, because the assumption of equal variance was not met, a one-way Welch ANOVA was used to evaluate the effect of noise levels on fGCM concentrations in NZP birds only, followed by a Games–Howell post hoc test. The data were log-transformed, and Levene’s test confirmed variance heterogeneity (*p* < 0.001). Six outliers were removed for this analysis.

## 4. Results

**Figure 1:**
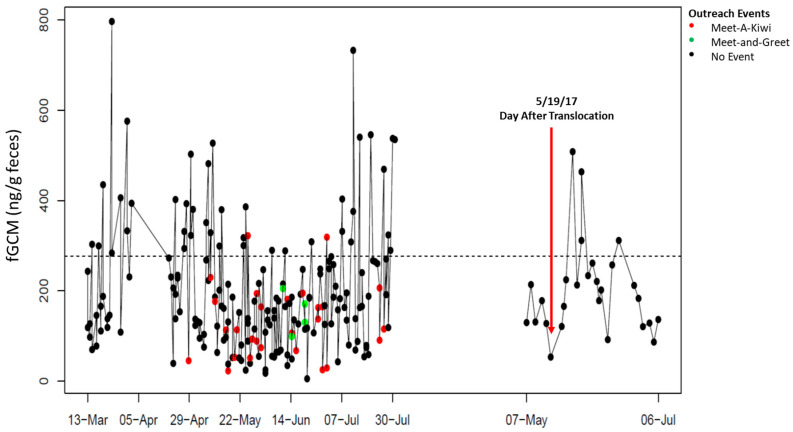
Longitudinal fecal glucocorticoid metabolite (fGCM) concentrations for ambassador bird NZP#1. NZP#1 was not translocated from NZP to SCBI on 18 May 2017. The red arrow denotes the fecal sample collected the day after translocation. Color markers denote ambassador events for NZP#1 (red: “Meet-A-Kiwi”; green: “Meet-and-Greet”; black: no event). The dotted line represents the hormone baseline calculated from the R package HormLong [44] using 1.5 standard deviations.

**Figure 2:**
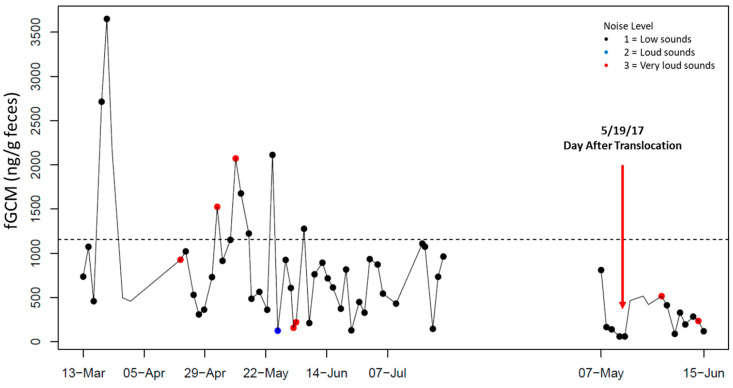
Longitudinal fecal glucocorticoid metabolite (fGCM) concentrations for control bird NZP#2. NZP#2 was translocated from NZP to SCBI on 18 May 2017. The red arrow denotes the fecal sample collected the day after translocation. Color markers denote noise levels noted by keepers at the end of the day for NZP birds (black: level 1, low sounds; blue: level 2, loud sounds; red: level 3, very loud sounds). The dotted line represents the hormone baseline calculated from the R package HormLong [44] using 1.5 standard deviations.

**Figure 3:**
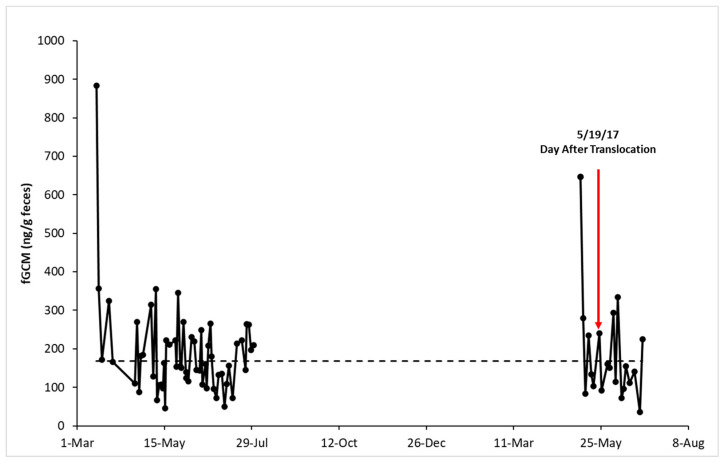
Longitudinal fecal glucocorticoid metabolite (fGCM) concentrations for control bird NZP#3. NZP#3 was translocated from NZP to SCBI on 18 May 2017. The red arrow denotes the fecal sample collected the day after the translocation event. The dotted line represents the hormone baseline calculated from the R package HormLong [44] using 1.5 standard deviations.

**Figure 4:**
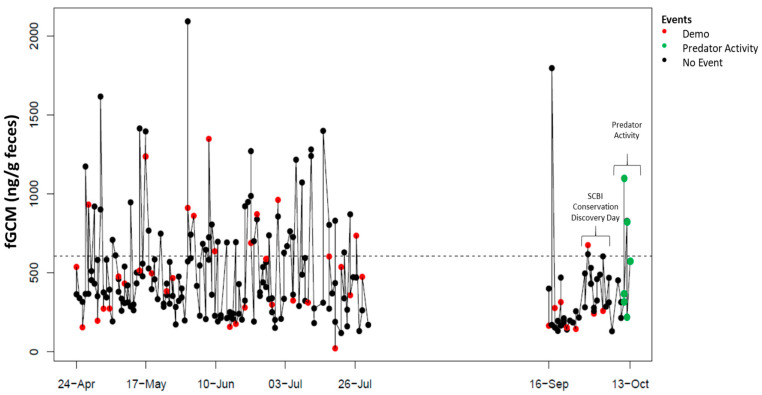
Longitudinal fecal glucocorticoid metabolite (fGCM) concentrations for ambassador bird SCBI#1. Color markers denote ambassador events for SCBI#1 (black: no event; red: demo event occurred). There was predator activity at the end of the study period that is noted in the profile. The dotted line represents the hormone baseline calculated from the R package, HormLong [44] using 1.5 standard deviations.

**Figure 5:**
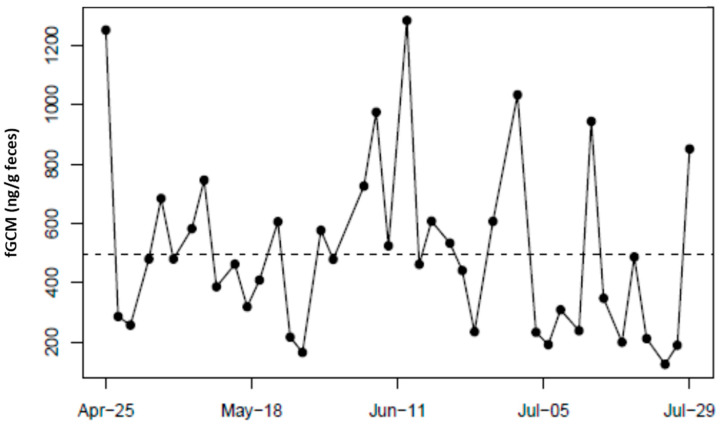
Longitudinal fecal glucocorticoid metabolite (fGCM) concentrations for control bird SCBI#2. SCBI#2 was the only female in the study. The dotted line represents the hormone baseline calculated from the R package HormLong [44] using 1.5 standard deviations.

Individual fGCM hormone profiles for NZP and SCBI birds are shown in Figure 1, Figure 2, Figure 3, Figure 4 and Figure 5, respectively. From October 10 to 14, 2016, keepers at SCBI noted predator activity around the outdoor enclosure of SCBI#1 (e.g., digging around fences and scat), which was reflected by an increase in fGCM concentrations (Figure 4).

**Figure 6:**
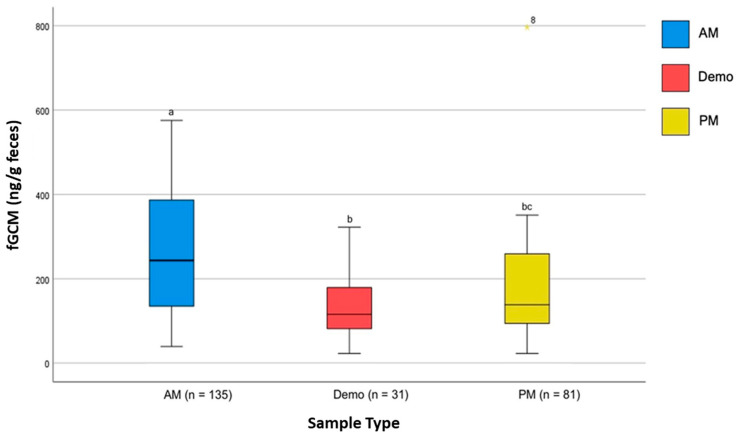
Effect of the sample type (AM, DEMO, and PM) on the mean fGCM concentrations of ambassador bird NZP#1. Within ambassador birds, the Friedman test revealed significant differences in fGCM concentrations for NZP#1 (*p* < 0.001) in the sample type. The post hoc analysis showed significantly higher fGCM concentrations during AM collection compared to DEMO and PM samples for NZP#1. a, b and c denote statistical significance at the 0.05 level. * is a high data point.

**Figure 7:**
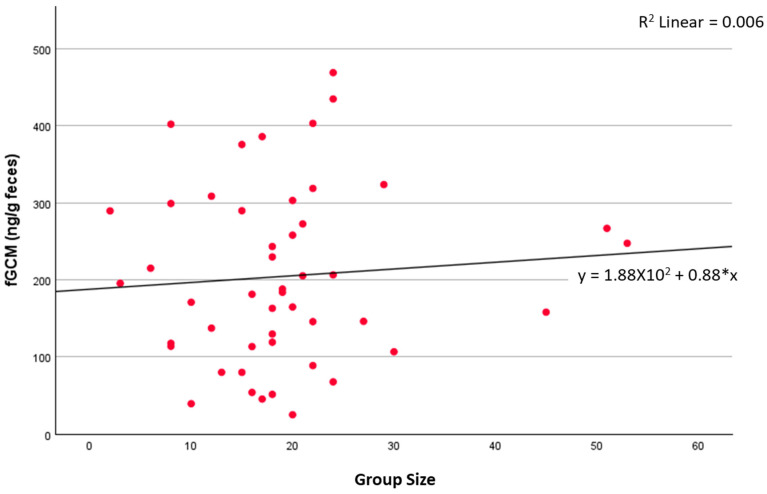
Number of attendees per event vs. fGCM concentrations for ambassador NZP#1. Meet-and-Greet group sizes ranged from 2 to 10 people, whereas demo audiences ranged from 8 to 100 people. An outlier of 100 people was removed and not shown.

Ambassador Status: A univariate analysis of variance found no significant interaction between the bird type (ambassador vs. control) and fGCM concentrations using AM samples as a unifying criterion. Within ambassador birds, the Friedman test revealed significant differences in fGCM concentrations for NZP#1 (*p* < 0.001), but not for SCBI#1 (*p* = 0.258) in the sample type. The post hoc analysis showed significantly higher fGCM concentrations during AM collection compared to DEMO and PM collections for NZP#1 (Figure 6). Group size did not significantly predict fGCM levels in the regression models (Figure 7).

**Figure 8:**
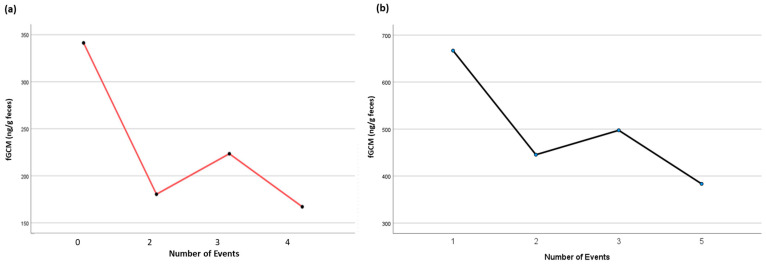
Effect of the number of events on the weekly mean fGCM concentrations for ambassador birds (**a**) NZP#1 and (**b**) SCBI#1. A pairwise comparison of fixed effects revealed significant differences between the specific number of events for NZP#1 (0 vs. 4 events, *p* = 0.021) and SCBI#1 (1 vs. 2 events, *p* = 0.047), with the mean fCGM concentrations decreasing as the event number increased for both birds.

Linear regression found no significant association between the event rate and fGCM concentrations for NZP#1 (F(1, 16) = 3.274, *p* = 0.089; outlier removed: F(1, 15) = 0.184, *p* = 0.674) or SCBI#1 (F(1, 16) = 1.604, *p* = 0.223). A linear mixed model analysis confirmed these findings, showing no significant fixed effects for NZP#1 (F(1, 3) = 142.152, *p* = 0.080) or SCBI#1 (F(1, 3) = 143.405, *p* = 0.185). However, a pairwise comparison of fixed effects revealed significant differences between specific number of events for NZP#1 (zero vs. four events, *p* = 0.021) and SCBI#1 (one vs. two events, *p* = 0.047), with mean fCGM concentrations decreasing as the event number increased for both birds (Figure 8).

**Figure 9:**
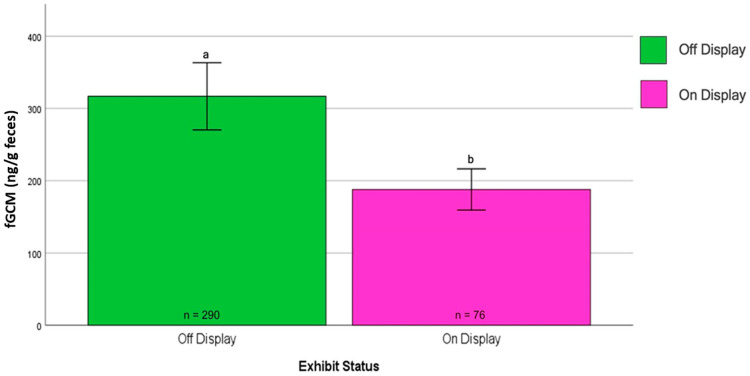
Effect of the display status on the mean fGCM concentrations for NZP birds. The Mann–Whitney U test revealed that birds on public display (pink bar) had significantly lower fGCM concentrations than off-display birds (green bar) (U = 9101, z = −2.34, *p* = 0.019). However, this difference was primarily driven by NZP#2, as the removal of this bird eliminated the significant difference. NZP#2 is included in this graphic. a and b denote statistical significance at the 0.05 level.

Facility and Display Status: The Kruskal–Wallis H test indicated significantly lower median fGCM concentrations at NZP (184.31 ng/g dry feces) than at SCBI (409.35 ng/g dry feces) (U = 76,671, z = 11.903, *p* < 0.001). Additionally, the Mann–Whitney U test revealed that birds on public display had significantly lower fGCM concentrations than off-display birds (U = 9101, z = −2.34, *p* = 0.019) (Figure 9). However, this difference was primarily driven by NZP#2, as the removal of this outlier eliminated the significant difference.

Age: Using AM samples, the Kruskal–Wallis H test showed significant differences in median fGCM concentrations among age groups (*p* < 0.001) at NZP but not at SCBI. NZP#2 (32 years old) exhibited significantly higher median AM fGCM levels than NZP#1 (5 years old) and NZP#3 (42 years old) (Table 3).

**Figure 10:**
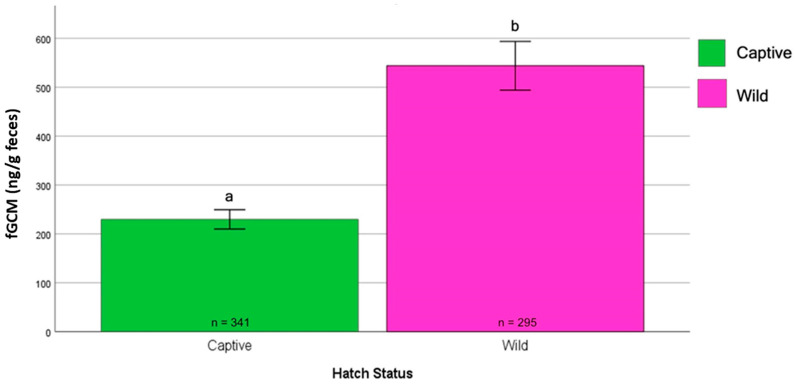
Effect of the hatching status on fGCM concentrations. Wild hatches (pink bar) included NZP#2 and SCBI#1, whereas captive hatches (green bar) included NZP#1, NZP#3, and SCBI#2. The Mann–Whitney U test indicated significantly higher median fGCM levels in wild-hatched birds (430.99 ng/g dry feces) compared to captive-hatched birds (182.18 ng/g dry feces) (U = 81,737, z = 13.605, *p* < 0.001). a and b denote statistical significance at the 0.05 level.

Hatching Status (Wild vs. Captive): The Mann–Whitney U test indicated significantly higher median fGCM levels in wild-hatched birds (430.99 ng/g dry feces) compared to captive-hatched birds (182.18 ng/g dry feces) (U = 81,737, z = 13.605, *p* < 0.001) (Figure 10).

**Figure 11:**
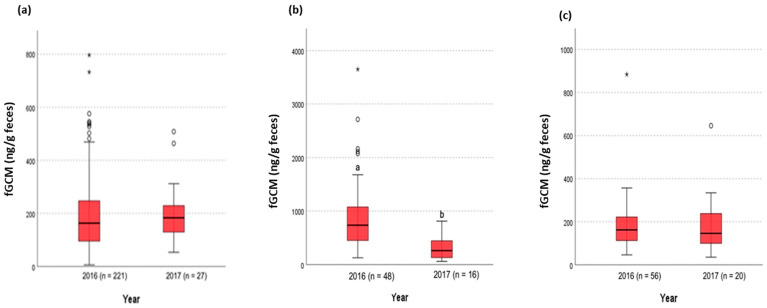
Effects of translocation on the fGCM concentrations of NZP birds. The Kruskal–Wallis H test demonstrated that (**b**) NZP#2 exhibited a significant decrease in fGCM concentrations after moving to SCBI (*p* < 0.001), while the other relocated bird (**a**) NZP#3 did not show significant changes (*p* = 0.547). The translocation control bird (**c**) NZP#1, which remained at NZP, also showed no significant changes (*p* = 0.202). a and b denote statistical significance at the 0.05 level. * are high data points.

Translocation: The Kruskal–Wallis H test demonstrated that NZP#2 exhibited a significant decrease in fGCM concentrations after moving to SCBI (*p* < 0.001), while the other relocated bird (NZP#3) did not show significant changes (*p* = 0.547). The control bird (NZP#1), which remained at NZP, also showed no significant changes (*p* = 0.202) (Figure 11).

**Figure 12:**
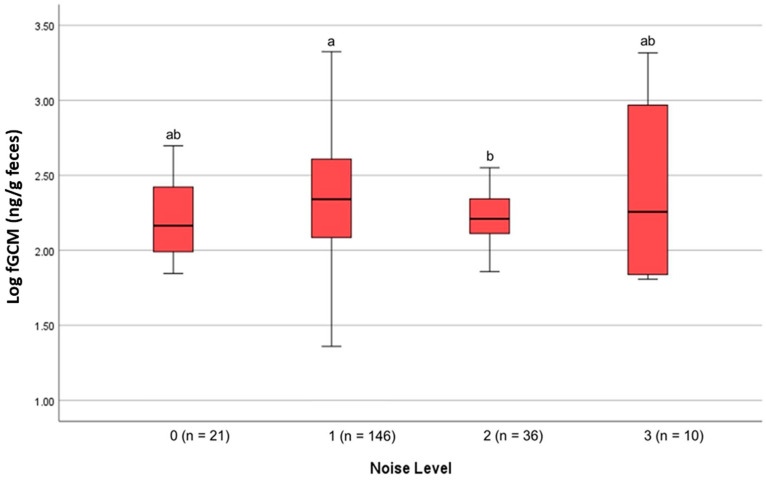
Effects of noise levels on the mean fGCM concentrations of NZP birds (N = 3). A one-way Welch ANOVA found significant differences in fGCM concentrations across noise levels (F(3, 32.062) = 2.998, *p* = 0.045). The post hoc analysis showed a significant increase in fGCM concentrations between noise levels 1 and 2 (*p* = 0.032). a and b denote statistical significance at the 0.05 level.

Noise Levels: A one-way Welch ANOVA found significant differences in fGCM concentrations across noise levels (F(3, 32.062) = 2.998, *p* = 0.045). The post hoc analysis showed a significant increase in fGCM concentrations between noise levels one and two (*p* = 0.032) (Figure 12).

## 5. Discussion

The objective of this study was to examine relationships between fGCM concentrations and factors such as environmental noise, outreach events, and translocation in brown kiwi housed at NZP and SCBI. The facility, age, hatching status, translocation, and noise disturbances significantly influenced fGCM levels, while the ambassador status, sample type, event rate, and public exposure had limited effects. These results underscore the complexity of generalizing GC responses to external stimuli in a captive environment and the need for longitudinal studies to better understand physiological responses over time. Such research is crucial to informing evidence-based management strategies that account for the unique physiology and natural history of species like the brown kiwi.

Our findings support the hypothesis that prior habituation of ambassador birds to outreach events resulted in no significant differences in fGCM concentrations compared to control birds. Similar to our findings, Cockrem [47] found that captive kiwi accustomed to handling and public display did not exhibit elevated plasma corticosterone levels. In that study, captive kiwi housed in nocturnal facilities or outdoor pens showed similar or lower corticosterone peaks compared to wild kiwi. Additionally, three regularly handled kiwi exhibited no corticosterone response when sampled after handling sessions, suggesting habituation to human interaction. In another study, Hartell-DeNardo et al. [48] demonstrated that ambassador Magellanic penguins (*Spheniscus magellanicus*) showed no significant differences in behavior or fGCM concentrations between weeks when they did or did not participate in outreach events, nor were there differences between penguins with 5 or 10 years of program experience. Interestingly, unlike in our study, they did not find significant individual variation in fGCM concentrations.

In this study, a gut transit time of 3 h was used based on the recommendations from the New Zealand Department of Conservation ([37], p. 44). The barium sulfate study that was conducted aligned well with this estimate. It is important to note that in the barium sulfate series, the bird was a juvenile and in a compromised state, both of which may have influenced the barium sulfate transit time. Additionally, barium sulfate transit does not directly reflect digestive transit, as barium sulfate is a non-absorbable suspension and likely moves through the gastrointestinal tract more rapidly than food. We collected fecal samples during and within 3 h of ambassador events to assess acute adrenal responses. Additionally, both AM and PM sampling were conducted for ambassador birds to account for potential lags in hormone excretion. In our study, we did not observe significant changes in fGCM concentrations before or after outreach events for either ambassador birds or compared to the control birds. Interestingly, our results suggest that ambassador kiwi birds may experience a reduced rather than heightened adrenal response to repeated outreach events, as fGCM concentrations decreased with more frequent events during the study period. Future studies with more frequent sampling could provide a clearer picture of the short-term adrenal responses in ambassador birds.

Ambassador kiwi birds may also perceive outreach events and human interaction as neutral rather than stressful, likely due to their high levels of habituation prior to the start of the study. Habituation, a widespread phenomenon, allows animals to filter out repetitive, non-threatening stimuli [49,50]. Recent studies suggest that habituation can be context-specific, involving associative learning where animals recognize certain stimuli within specific environments [50,51]. Similar learning patterns have been observed in precocial birds like domestic fowl, which, like brown kiwi, are cognitively and functionally developed at hatching. Studies show that young domestic fowl exhibit context-specific habituation, learning to associate certain sounds with specific surroundings. For instance, chicks exposed to repetitive sounds in a stable environment reduced their startle response over time, while those experiencing environmental changes maintained a heightened startle response to the same sound [52].

We propose that ambassador kiwi may similarly associate outreach events with neutral or positive outcomes due to early exposure to repetitive, non-threatening stimuli (i.e., habituation) with their primary keepers. This capacity for context-specific learning may help them remain calm and adaptable during events. In a study with African penguins (*Spheniscus demersus*), Scheun et al. [53] investigated the effect of tourism on the urofecal GC metabolite (ufGCM) concentrations of wild chicks from two breeding colonies. Researchers found that penguin chicks experiencing low, infrequent human disturbance had significantly higher mean ufGCM concentrations compared to chicks with exposure to moderate or high levels of tourism. The authors concluded that the African penguin chicks that were exposed to frequent human activity may have been habituated to tourism and thus had comparatively lower ufGCM concentrations. Future research should examine how early habituation influences behavioral and physiological responses in brown kiwi, comparing ambassador and non-ambassador birds to better understand the cognitive mechanisms behind their adaptability.

We examined whether fGCM concentrations varied by sample type (AM, PM, and DEMO) for ambassador birds. No significant differences were found across sample types or between birds, though individual variation was noted. For example, NZP#1 had higher fGCM concentrations in AM samples that were collected in the early morning, possibly reflecting a lag in GC excretion from the previous night. Diurnal GC rhythms are well-documented in birds, with plasma GC levels often peaking before active periods [54,55]. In nocturnal screech owls (*Megascops choliba*), fGCM concentrations were highest in the evening, aligning with increased activity [56]. Similarly, the elevated AM fGCM concentrations of NZPI#1 may represent delayed metabolite excretion representative of a nighttime increase rather than a response to ambassador events [30], while the lower fGCM concentrations observed in samples collected throughout the day was reflective of normal fluctuations in this nocturnal species. Without repeated sampling in control birds during the day, we cannot confirm this nocturnal pattern, but it warrants further investigation to clarify the GC dynamics in brown kiwi.

We hypothesized that birds housed at the NZP, particularly the bird on public display (NZP#3), would exhibit higher fGCM concentrations compared to birds housed at SCBI, which are kept in large, naturalistic enclosures with limited human contact. However, our findings contradicted this hypothesis, as birds at NZP had significantly lower fGCM concentrations than those at SCBI. Among the NZP birds, the individual on public display (NZP#3) exhibited significantly lower fGCM concentrations than the off-display birds (NZP#1 and NZP#2). Notably, this result was largely driven by NZP#2, which displayed elevated fGCM concentrations. NZP#2 was neither an ambassador bird nor on public display, suggesting that individual variation played a prominent role in this study.

Individual variation was particularly evident in our analysis of the translocation of two NZP birds to SCBI in 2017. We initially hypothesized that translocated birds would exhibit a short-term increase in the adrenal GC response; however, this was not observed. Notably, NZP#2, which had the highest fGCM concentrations while at NZP, experienced a significant decrease in fGCM concentrations after the move. Similar patterns of varied physiological responses to translocation have been observed in other species. In a study of chukar partridge (*Alectoris chukar*), researchers simulated a translocation event involving capture, handling, temporary holding, and release into an unfamiliar environment [57]. Their findings revealed a reduced adrenal response in translocated birds, as evidenced by lower blood corticosterone levels compared to control individuals [57]. Likewise, a study of greater stick-nest rats (*Leporillus conditor*) examined post-translocation changes in fGCMs across three source populations: remnant-wild, reintroduced-wild, and captive-bred [58]. fGCMs remained stable in the remnant-wild population, decreased significantly in the reintroduced-wild population, and increased significantly in the captive-bred population [58]. Interestingly, NZP#2 was the only wild-hatched bird that was moved.

In a related study, Leche et al. [38] investigated fGCM concentrations in 20 Greater Rhea (*Rhea americana*) translocated from captivity to a wildlife refuge. They observed an increase in fGCM levels one day after transport, followed by a return to baseline shortly thereafter [38]. NZP#1 (who did not undergo an translocation in 2017) and NZP#3 showed no significant changes. Interestingly, neither the ambassador bird (NZP#1) nor the public display bird (NZP#3) exhibited changes in fGCM concentrations after the translocation event. For NZP#3, this move included going into a larger enclosure with reduced anthropogenic disturbance. It is also possible that the absence of an immediate spike in fGCM concentrations post-translocation in our study was due to the relatively short gut transit time in kiwi, which may have limited our ability to detect acute responses with only daily fecal sampling [30,33]. The variation in individual responses highlights the need for further research into species-specific and individual physiological responses to environmental changes. Translocations play a crucial role in the conservation and management of rare and threatened species. Incorporating physiological measures, such as fecal GC metabolites, have the potential to enhance translocation success and fine-tune management practices.

Noise levels at NZP did not appear to influence hormone production in the studied birds. Similar findings have been reported in other avian species, such as European starlings, tree swallows, zebra finches, and house wrens, which showed no significant corticosterone elevations in response to noise disturbance [59]. However, in a related study, emus exhibited elevated corticosterone concentrations during loud construction noise [60]. Meanwhile, Kleist et al. [61] found that increased anthropogenic noise disturbance was associated with decreased baseline corticosterone levels in adults and nestling western (*Sialia mexicana*) and mountain (*Sialia currucoides*) bluebirds. Taken together, our findings suggest that the brown kiwi at NZP were likely acclimated to their consistent and predictable environment and occasional above-normal noise disturbances.

Another potential explanation for the higher fGCM concentrations observed in SCBI birds is the environmental context. Although SCBI birds are supplemented with food, they engage in active foraging within their large outdoor enclosures (W. Bailey, unpublished [11]). This behavior may result in physiological responses more similar to those of wild kiwi. Supporting this hypothesis, Adams [62] demonstrated that wild northern brown kiwi had significantly higher blood cortisol concentrations compared to captive individuals. Meanwhile, captive kiwi populations exhibited lower basal cortisol concentrations and no evidence of chronic stress [62]. In a study of Macaroni penguins (*Eudyptes chrysolophus*), Crossin et al. [63] found that increased plasma corticosterone levels were associated with higher levels of foraging and diving activity in females that were treated with exogenous corticosterone implants. The authors hypothesize that corticosterone supports foraging activity in this species. Similarly, Angelier et al. [64] found that plasma corticosterone levels were positively associated with the daily distance traveled and maximum range at sea in wandering albatrosses (*Diomedea exulans*). The elevated fGCM concentrations in SCBI birds may, therefore, reflect an adaptive physiological response to the increased energy demands associated with naturalistic behaviors, such as foraging, nest building, and territorial maintenance, rather than being indicative of chronic stress.

Providing large, naturalistic enclosures that promote species-specific behaviors and minimize human disturbance aligns with best practices for avian husbandry [65]. Such practices are especially relevant for breeding programs housing endangered species, such as SCBI’s kiwi breeding facility. These environments and the corresponding physiological responses they elicit may support the overall health and reproductive success of brown kiwi by mimicking conditions experienced in the wild. Further research is needed to fully understand the range of physiological responses of brown kiwi to captive environments and breeding facilities. This could include exploring the interplay of individual variation, environmental factors, and behavioral adaptations to better inform conservation and husbandry practices for this species.

Our data found that wild-hatched birds (NZP#2 and SCBI#1) exhibited higher GC responses compared to their captive-hatched counterparts, irrespective of the facility. Similar findings have been reported in a larger study of brown kiwi by Cockrem [47], who observed significantly higher GC concentrations in wild-hatched kiwi birds compared to captive-hatched individuals. The elevated fGCM responses in wild-hatched birds could reflect physiological or behavioral differences linked to their early-life experiences in the wild, including exposure to stressors such as predators, competition, or environmental variability [47]. These early-life experiences may lead to long-term changes in stress physiology, even after years in captivity. Further research investigating the potential effects of hatching origin on the stress physiology, behavior, and overall health of kiwi could provide valuable insights for conservation and captive management programs.

## 6. Conclusions

This study is the first to measure fGCM concentrations in brown kiwi under human care. The results indicate that the fGCM analysis effectively captures physiological responses to environmental changes. An acute increase in fGCM levels following predator activity near the enclosure of ambassador bird SCBI#1 confirms the assay’s ability to detect short-term stress responses. The absence of similar spikes before or after ambassador events suggests distinct physiological responses to different environmental stimuli, highlighting the need for further research to identify factors that optimize the welfare of this species. Translocation effects were also evident, as one bird exhibited a significant decrease in fGCM concentrations following relocation from NZP to SCBI. Additionally, significant differences in fGCM levels between wild-hatched and captive-hatched birds align with previous research [40] reporting similar GC variations using blood samples. This consistency with prior studies further supports the validity of the fGCM analysis in detecting biologically meaningful hormonal differences among the study birds.

Our findings have important implications for the captive management of brown kiwi, particularly in ambassador programs and breeding facilities. The observed differences in fGCM concentrations based on the facility, hatching status, and translocation highlight the need for individualized management approaches that account for an animal’s background and environmental context. The absence of significant stress responses in ambassador kiwi suggests that well-structured habituation protocols can effectively prepare birds for outreach activities without compromising their welfare. Additionally, the higher fGCM concentrations observed in birds housed in large, naturalistic enclosures at SCBI may reflect increased energy demands rather than chronic stress, emphasizing the need to balance enclosure design with behavioral and physiological needs. These results reinforce the importance of species-specific husbandry strategies that integrate both controlled and naturalistic environments to support the overall health and well-being of captive kiwi. Future research should explore how long-term exposure to different management practices influences physiological and behavioral outcomes to refine best practices for kiwi conservation and care. Additionally, our study could not assess seasonal or circadian variations in GC secretion in this species. Future studies that collect samples for at least one year, with increased sampling throughout the day, would enhance our understanding of GC dynamics in this species. This will aid in refining management strategies and welfare practices, particularly for birds in ambassador programs.

## Figures and Tables

**Table 1:** Study animal demographics. This table summarizes demographic and housing information for the five birds included in the study, categorized by their roles as ambassadors or controls. The data include individual identifiers, age, sex, origin (captive- or wild-hatched), housing conditions (indoor or outdoor), exhibit status (on or off display), collection dates, translocation status (yes or no), and total number of fecal samples collected.

Bird ID	Bird Type	Age	Sex	Hatch	Housing	Exhibit Status	Collection Dates	Translocated in 2017	Sample Total
NZP#1	Ambassador	5	M	Captive	Outdoors	Off	14 March 2016–1 August 2016, 8 May 2017–7 July 2017	No	221
NZP#2	Control	32	M	Wild	Outdoors	Off	14 March 2016–29 July 2016, 8 May 2017–16 June 2017	Yes	49
NZP#3	Control	42	M	Captive	Indoors	On	14 March 2016–29 July 2016, 8 May 2017–30 June 2017	Yes	76
SCBI#1	Ambassador	46	M	Wild	Outdoors	Off	25 April 2016–31 July 2016, 17 September 2016–14 October 2016	No	229
SCBI#2	Control	4	F	Captive	Outdoors	Off	25 April 2016–29 July 2016	No	40

**Table 2:** NZP noise level definitions. This table defines a four-level scale for categorizing the environmental noise intensity at SNZP, ranging from no discernable sounds (Level 0) to very loud sounds caused by equipment or heavy activity (Level 3). Intermediate levels include low ambient sounds (Level 1) and loud but non-mechanical sounds like clapping or music (Level 2).

Noise Level	Definition	Number of Samples
0	No discernable noise	21
1	Low noise, such as talking or traffic noise from a road	146
2	Loud noise, such as yelling, clapping, or music	36
3	Very loud noise, such as noisy equipment, mowing, leaf blowing, banging on glass, electric hedge trimmers, or generators	10

**Table 3:** Descriptive summary measures of fecal glucocorticoid metabolites (fGCMs) for the study population (N = 5). The data are organized by bird ID, sample type (AM, PM, Demo), and bird type (ambassador, control). Descriptive measures include means, medians, standard errors of the means (SEMs), percent coefficient of variation (percent CV), minimum (Min), maximum (Max), baseline, and base and peak means.

Bird ID	Age	Sample Type	Bird Type	n	Mean (ng/mg Dry Feces)	Median (ng/mg Dry Feces)	SEM	Percent CV	Min (ng/mg Dry Feces)	Max (ng/mg Dry Feces)	Baseline (ng/mg Dry Feces)	Base Mean (ng/mg Dry Feces)	Peak Mean (ng/mg Dry Feces)
NZP#1		AM	Ambassador	108	205.46	184.19 ^b^	12.15	61.48	17.72	575.52	171.52	104.54	299.17
NZP#1	5	PM		82	179.52	139.06	15.65	78.93	5.48	796.17	144.34	87.4	276.25
NZP#1		Demo		31	133.92	115.53	13.81	57.43	22.89	322.12	123.72	74.44	197.36
NZP#2	32	AM	Control	49	880.86	734.95 ^a^	99.56	79.12	125.88	3650.29	618.85	380.74	1288.37
NZP#3	42	AM	Control	76	187.85	155.71 ^b^	14.35	66.60	35.36	884.21	167.97	116.02	281.44
SCBI#1		AM	Ambassador	108	434.30	347.87	25.54	61.10	118.34	1413.83	377.08	266.75	678.01
SCBI#1	46	PM		76	566.53	463.97	46.34	71.30	130.92	2092.01	283.47	213.49	701.33
SCBI#1		Demo		45	486.12	459.94	43.28	59.72	21.51	1347.93	542.22	331.74	827.94
SCBI#2	4	AM	Control	40	503.87	471.01	46.22	58.01	125.75	1283.57	497.51	317.58	783.30

^ab^ Significantly different based on a Kruskal–Wallis H test for age effects within NZP at *p* < 0.05.

## Data Availability

The data are available upon request from the communicating author, W. Bailey.

## Supplementary Materials

The following supporting information can be downloaded at https://www.mdpi.com/article/10.3390/ani15081156/s1: Photo S1. Picture of an ambassador bird with KB during a “Meet-A-Kiwi” event; Photo S2. Picture of an ambassador bird in a demonstration box during an outreach event; Photo S3. Ambassador bird with NAP during a mock VIP event; Photo S4. Ambassador bird SCBI#1 during Conservation Discovery Day Event in October 2016; Image S1. X-ray image at 95 min after the oral administration of barium sulfate; Image S2. X-ray image at 305 min after the oral administration of barium sulfate; and Image S3. X-ray image at 23 h after the oral administration of barium sulfate).

## References

[1] Department of Conservation (2020). Brown Kiwi. *Kiwi*. www.doc.govt.nz/nature/native-animals/birds/birds-a-z/kiwi/brown-kiwi/.

[2] Holzapfel S., Robertson H.A., McLennan J.A., Sporle W., Hackwell K., Impey M. (2008). *Kiwi (Aptryx* ssp.*) Recovery Plan 2008-2018*. Retrieved from Wellington, New Zealand. https://www.doc.govt.nz/Documents/science-and-technical/tsrp60entire.pdf.

[3] Robertson H.A., Baird K., Dowding J.E., Elliott G.P., Hitchmough R.A., Miskelly C.M., Taylor G.A. (2017). *Conservation Status of New Zealand Birds, 2016*. Retrieved from Wellington, New Zealand. https://www.doc.govt.nz/documents/science-and-technical/nztcs19entire.pdf.

[4] Robertson H.A., Colbourne R.M., Graham P.J., Miller P.J., Pierce R.J. (2011). Experimental management of Brown kiwi *Apteryx mantelli* in central Northland, New Zealand. Bird Conserv. Int..

[5] Bryan C.G., Brader K. (2019). North Island Brown Kiwi (Apteryx Australis Mantelli) AZA Species Survival Plan—Yellow Program.

[6] McLennan J.A., McCann A.J. (1991). Short communication—Incubation temperatures of great spotted kiwi, *Apteryx haastii*. N. Z. J. Ecol..

[7] Kinsky F.C. (1971). The consistent presence of paired ovaries in the kiwi (*Apteryx*) with some discussion of this condition in other birds. J. Ornithol..

[8] Cunningham S.J., Castro I. (2011). The secret life of wild brown kiwi: Studying behaviour of a cryptic species by direct observation. N. Z. J. Ecol..

[9] Colbourne R. (2002). Incubation behaviour and egg physiology of kiwi (*Apteryx* spp.) in natural habitats. N. Z. J. Ecol..

[10] Calder W.A. (1978). The Kiwi. Sci. Am..

[11] Bailey W. (Smithsonian National Zoo and Conservation Biology Institute, Front Royal, VA, USA).

[12] French F., Bwye P., Carrigan L., Coe J.C., Kelly R., Leek T., Lynch E.C., Mahan E., Mingee C. (2024). Welfare and Enrichment of Managed Nocturnal Species, Supported by Technology. Animals.

[13] Fernandez E.J., Tamborski M.A., Pickens S.R., Timberlake W. (2009). Animal-visitor interactions in the modern zoo: Conflicts and interventions. Appl. Anim. Behav. Sci..

[14] Jensen T., Durrant B. (2006). Assessment of reproductive status and ovulation in female brown kiwi (*Apteryx mantelli*) using fecal steroids and ovarian follicle size. Zoo Biol..

[15] Colbourne R., Bassett S., Billing T., McCormick H., McLennan J., Nelson A., Robertson H. (2005). The Development of Operation Nest Egg as a Tool in the Conservation Management of Kiwi.

[16] Brader K. (Smithsonian National Zoo and Conservation Biology Institute, Washington, DC, USA).

[17] Smulders T.V. (2021). Telencephalic regulation of the HPA axis in birds. Neurobiol. Stress.

[18] Cockrem J.F. (2007). Stress, corticosterone responses and avian personalities. J. Ornithol..

[19] Sapolsky R.M., Romero L.M., Munck A.U. (2000). How do glucocorticoids influence stress responses? Integrating permissive, suppressive, stimulatory, and preparative actions. Endocr. Rev..

[20] Wingfield J.C., Breuner C.W., Jacobs J., Harvey S., Etches R.J. (1997). Corticosterone and behavioral responses to unpredictable events. Perspectives in Avian Endocrinology.

[21] Wingfield J.C., Ramenofsky R., Balm P.H.M. (1999). Hormones and the behavioral ecology of stress. Stress Physiology in Animals.

[22] Landys M.M., Ramenofsky M., Wingfield J.C. (2006). Actions of glucocorticoids at a seasonal baseline as compared to stress-related levels in the regulation of periodic life processes. Gen. Comp. Endocrinol..

[23] Munck A., Guyre P.M., Holbrook N.J. (1984). Physiological functions of glucocorticoids in stress and their relation to pharmacological actions. Endocrinol. Rev. Winter.

[24] Wingfield J.C., Kitaysky A.S. (2002). Endocrine Responses to Unpredictable Environmental Events: Stress or Anti-Stress Hormones?. Integr. Comp. Biol..

[25] Silverin B. (1986). Corticosterone-binding proteins and behavioral effects of high plasma levels of corticosterone during the breeding period in the pied flycatcher. Gen. Comp. Endocrinol..

[26] Cockrem J., Silverin B. (2002). Variant within and between birds in corticosterone responses of great tits (*Parus major*). Gen. Comp. Endocrinol..

[27] Fraisse F., Cockrem J.F. (2006). Corticosterone and the measurement of stress and fear in cage housed laying chickens. Br. Poult. Sci..

[28] Satterlee D.G., Johnson W.A. (1986). Selection of Japanese quail for contrasting blood corticosterone response to immobilization. Poult. Sci..

[29] Goymann W. (2005). Noninvasive monitoring of hormones in bird droppings: Physiological validation, sampling, extraction, sex differences, and the influence of diet on hormone metabolite levels. Ann. N. Y. Acad. Sci..

[30] Palme R., Rettenbacker S., Touma C., El-Bahr S.M., Mostl E. (2006). Stress hormones in mammals and birds: Comparative aspects regarding metabolism, excretion, and non-invasive measurement in fecal samples. Ann. N. Y. Acad. Sci..

[31] Carere C., Groothuis T.G.G., Möstl E., Daan S., Koolhaas J.M. (2003). Fecal corticosteroids in a territorial bird selected for different personalities: Daily rhythm and the response to social stress. Horm. Behav..

[32] Hirschenhauser K., Kotrschal K., Möstl E. (2005). Synthesis of measuring steroid metabolites in goose feces. Ann. N. Y. Acad. Sci..

[33] Morgan K.J. (2008). Kiwi First Aid and Veterinary Care. Department of Conservation. https://www.doc.govt.nz/documents/science-and-technical/sap245entire.pdf.

[34] Sales J. (2006). Digestive physiology and nutrition of ratites. Avian Poult. Biol. Rev..

[35] Herd R.M., Dawson T.J. (1984). Fibre digestion in the emu, *Dromaius novaehollandiae*, a large bird with a simple gut and high rate of passage. Physiol. Zool..

[36] Wilson M.F. (1989). Gut retention times of experimental pseudoseeds by emus. Biotropica.

[37] Westcott D.A., Bentrupperbäumer J., Bradford M.G., McKeown A. (2005). Incorporating patterns of disperser behaviour into models of seed dispersal and its effects on estimated dispersal curves. Oecologia.

[38] Leche A., Vera Cortez M., Della Costa N.S., Navarro J.L., Marin R.H., Martella M.B. (2016). Stress response assessment during translocation of captive-bred Greater Rheas into the wild. J. Ornithololgy.

[39] Stewart J., Ritchie B.W., Harrison G.J., Harrison L.R. (1994). Ratites. Avian Medicine: Principles and Applications.

[40] Watson R., Munro C., Edwards K.L., Norton V., Brown J.L., Walker S.L. (2013). Development of a versatile enzyme immunoassay for non-invasive assessment of glucocorticoid metabolites in a diversity of taxonomic species. Gen. Comp. Endocrinol..

[41] Wasser S.K., Hunt K.E., Brown J.L., Cooper K., Crockett C.M., Bechert U., Millspaugh J.J., Larson S., Monfort S.L. (2000). A generalized fecal glucocorticoid assay for use in a diverse array of nondomestic mammalian and avian species. Gen. Comp. Endocrinol..

[42] Donelan E.M., Philpott M.P., MacKinnon K.M., Klosterman K.A., Roth T.L. (2022). Fecal glucocorticoid metabolite concentrations associated with illness, sex, age, and season in a kea *Nestor notabiliis* population at the Cincinnati Zoo and Botanical Garden. J. Zoo Aquar. Res..

[43] Brown J.L., Wildt D.E., Wielebnowski N., Goodrowe K.L., Graham L.H., Wells SHoward J.G. (1996). Reproductive activity in captive female cheetahs (*Acinonyx jubatus*) assessed by faecal steroids. J. Reprod. Fertil..

[44] Fanson B., Fanson K. (2014). hormlong: Longitudinal Analysis of Hormone Data. R Package, Version 2.13.2. https://www.researchgate.net/publication/308965751_hormLong_An_R_package_for_longitudinal_data_analysis_in_wildlife_endocrinology_studies.

[45] Greenhouse S.W., Geisser S. (1959). On the methods in the analysis of profile data. Psychometrika.

[46] Dunn O.J. (1964). Multiple comparisons using rank sums. Technometrics.

[47] Cockrem J., Adams D., Bennett E., Candy E., Henare S., Hawke E., Potter M., Gordon M.S., Bartol S.M. (2004). Endocrinology and the conservation of New Zealand birds. Experimental Approaches to Conservation Biology.

[48] Hartell-DeNardo J., Kozlowski C., Baskir E., Macek M., Dorsey C., Powell D.M. (2023). Behavior and adrenal physiology of Magellanic penguins (*Spheniscus magellanicus*) serving as ambassador animals. Zoo Biol..

[49] Thompson R.F. (2009). Habituation: A history. Neurobiol. Learn. Mem..

[50] Dissegna A., Turatto M., Chiandetti C. (2021). Context-specific habituation: A review. Animals.

[51] McDiarmid T.A., Yu A.J., Rankin C.H. (2019). Habituation Is More Than Learning to Ignore: Multiple Mechanisms Serve to Facilitate Shifts in Behavioral Strategy. Bioessays.

[52] Chiandetti C., Turatto M. (2017). Context-specific habituation of the freezing response in newborn chicks. Behav. Neurosci..

[53] Scheun J., Miller R.J., Ganswindt A., Waller L.J., Pichegru L., Sherley R.B., Maneveldt G.W. (2021). Urofaecal glucocorticoid metabolite concentrations in African penguin (*Sheniscus demersus*) chick populations experiencing different levels of human disturbance. Conserv. Physiol..

[54] Oster H., Challet E., Ott V., Arvat E., de Kloet E.R., Dijk D., Lightman S., Vgontzas A., Van Cauter E. (2017). The functional and clinical significance of the 24-h rhythm of circulating glucocorticoids. Endocr. Rev..

[55] Spiga F., Walker J.J., Terry J.R., Lightman S.L. (2014). HPA axis-rhythms. Compr. Physiol..

[56] Barbosa-Moyano H., Sobral G., de Oliveira C.A. (2023). Glucocorticoid metabolites in an ex-situ nocturnal bird, the tropical screech owl *Megascops choliba*: Effects of sex, activity period, and inter-individual variation. Conserv. Physiol..

[57] Dickens M.J., Delehanty D.J., Romero L.M. (2009). Stress and translocation: Alterations in the stress physiology of translocated birds. Proceedings. Biol. Sci..

[58] Williams-Kelly K.S., Berry L., Branch K., Cowen S., Garretson S., Holland G.J., Ladd R., Olds L., Rayner K., Sims C. (2023). Physiological response after translocation differs between source populations in a threatened mammal. R. Soc. Open Sci..

[59] Injaian A.S., Francis C.D., Ouyang J.Q., Dominoni D.M., Donald J.W., Fuxjager M.J., Goymann W., Hau M., Husak J.F., Johnson M.A. (2020). Baseline and stress-induced corticosterone levels across birds and reptiles do not reflect urbanization levels. Conserv. Physiol..

[60] Jakob-Hoff R., Kingan M., Chiaki F., Schmid G., Cockrem J.F., Crackle A., Van Bemmel E., Connor R., Descovich K. (2019). Potential Impact of Construction Noise on Selected Zoo Animals. Hum. Influ. Behav. Welf. Zoo Anim..

[61] Kleist N.J., Guralnick R.P., Cruz A., Lowry C.A., Francis C.D. (2018). Chronic anthropogenic noise disrupts glucocorticoid signaling and has multiple effects on fitness in an avian community. Proc. Natl. Acad. Sci. USA.

[62] Adams D.C. (2000). Corticosterone Responses of Captive and Wild Brown Kiwi (*Apteryx Mantelli*): A Thesis Presented in Partial Fulfillment of the Requirements for the Degree of Master of Science in Zoology. Master’s of Science.

[63] Crossin G.T., Trathan P.N., Phillips R.A., Gorman K.B., Dawson A., Sakamoto K.Q., Williams T.D. (2012). Corticosterone predicts foraging behavior and parental care in Macaroni Penguins. Am. Nat..

[64] Angelier F., Shaffer S.A., Weimerskirch H., Trouve C., Chastel O. (2007). Corticosterone and foraging behavior in a pelagic seabird. Physiol. Biochem. Zool..

[65] Rose P., Freeman M., Hickey I., Kelly R., Greenwell P. (2024). Considering what animals “need to do” in enclosure design: Questions for bird flight and aviaries. Birds.

