# Supplementary figures and images for: Fecal Glucocorticoid Metabolite Responses of Brown Kiwi (Apteryx mantelli) to Ambassador Program Participation and Translocation: Implications for Captive Management and Welfare
Source: Animals (Basel). 2025 Apr 17;15(8):1156. doi: 10.3390/ani15081156 (PMC12024267; doi:10.3390/ani15081156)

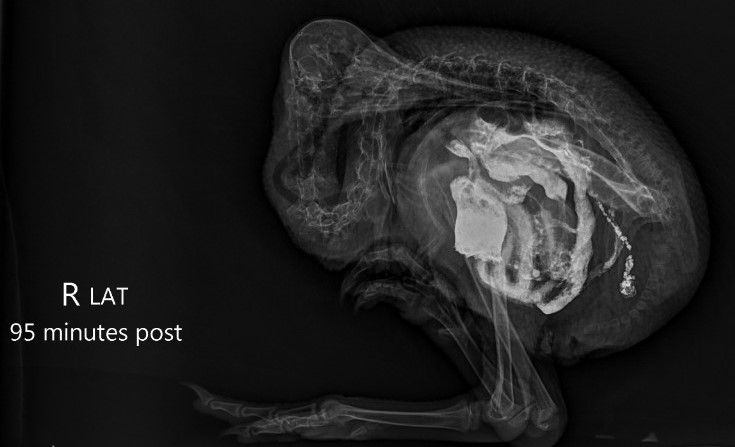

Supplement: Supplementary file 1 [file animals-15-01156-s001.zip › animals-3492583-supplementary/Image 1 - Kiwi 2016-09-03 95 min post barium.jpg]

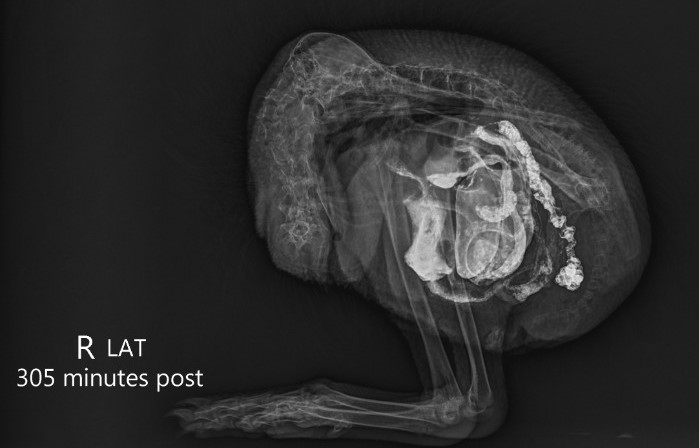

Supplement: Supplementary file 1 [file animals-15-01156-s001.zip › animals-3492583-supplementary/Image 2-Kiwi 2016-09-03 305 min post barium.jpg]

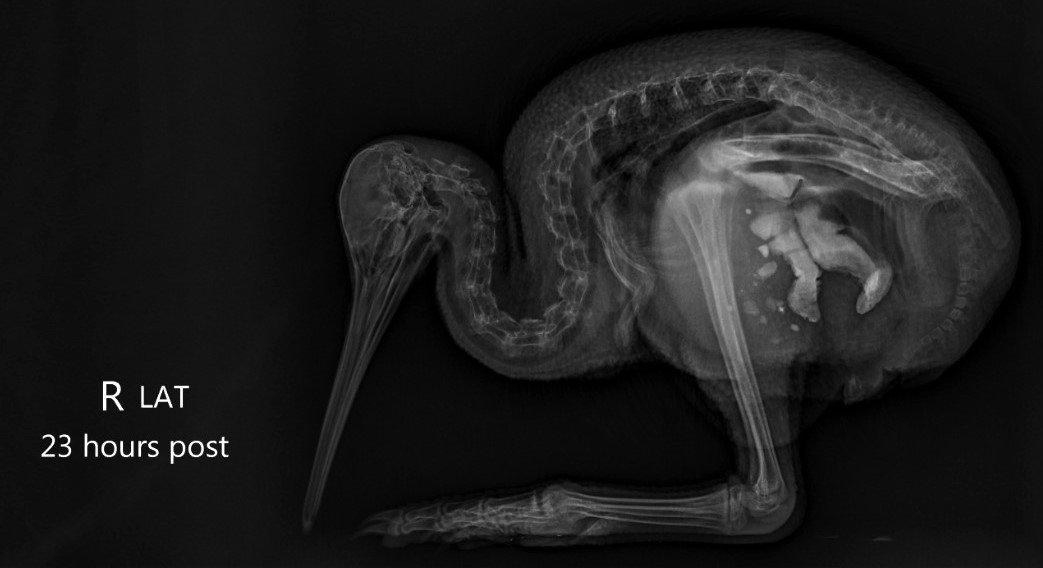

Supplement: Supplementary file 1 [file animals-15-01156-s001.zip › animals-3492583-supplementary/Image 3-Kiwi 2016-09-04 23 hrs post barium.jpg]

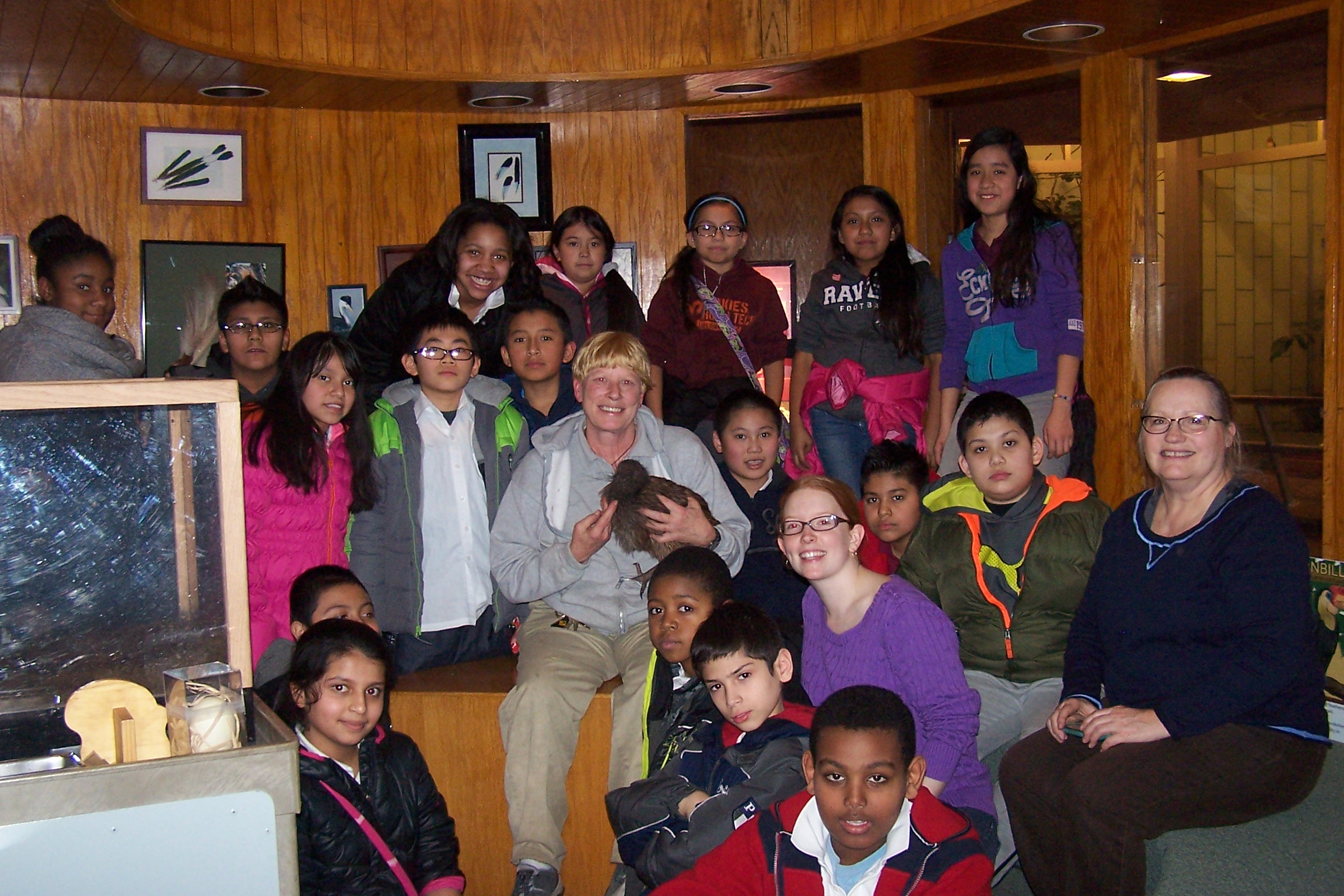

Supplement: Supplementary file 1 [file animals-15-01156-s001.zip › animals-3492583-supplementary/Supplementary Photo #1.JPG]

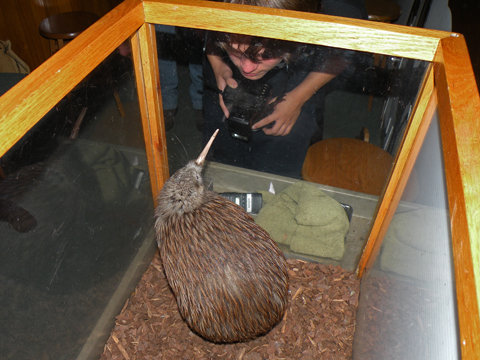

Supplement: Supplementary file 1 [file animals-15-01156-s001.zip › animals-3492583-supplementary/Supplementary Photo #2.jpg]

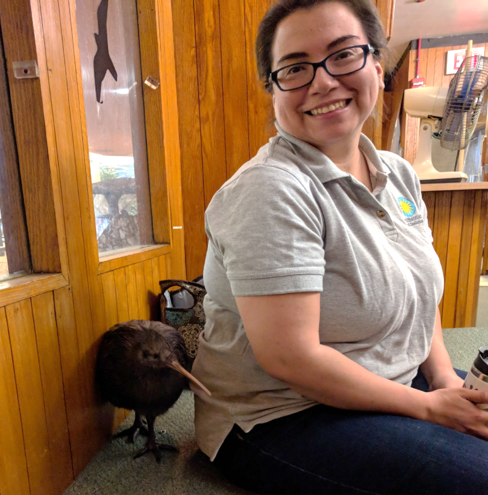

Supplement: Supplementary file 1 [file animals-15-01156-s001.zip › animals-3492583-supplementary/Supplementary Photo #3.png]

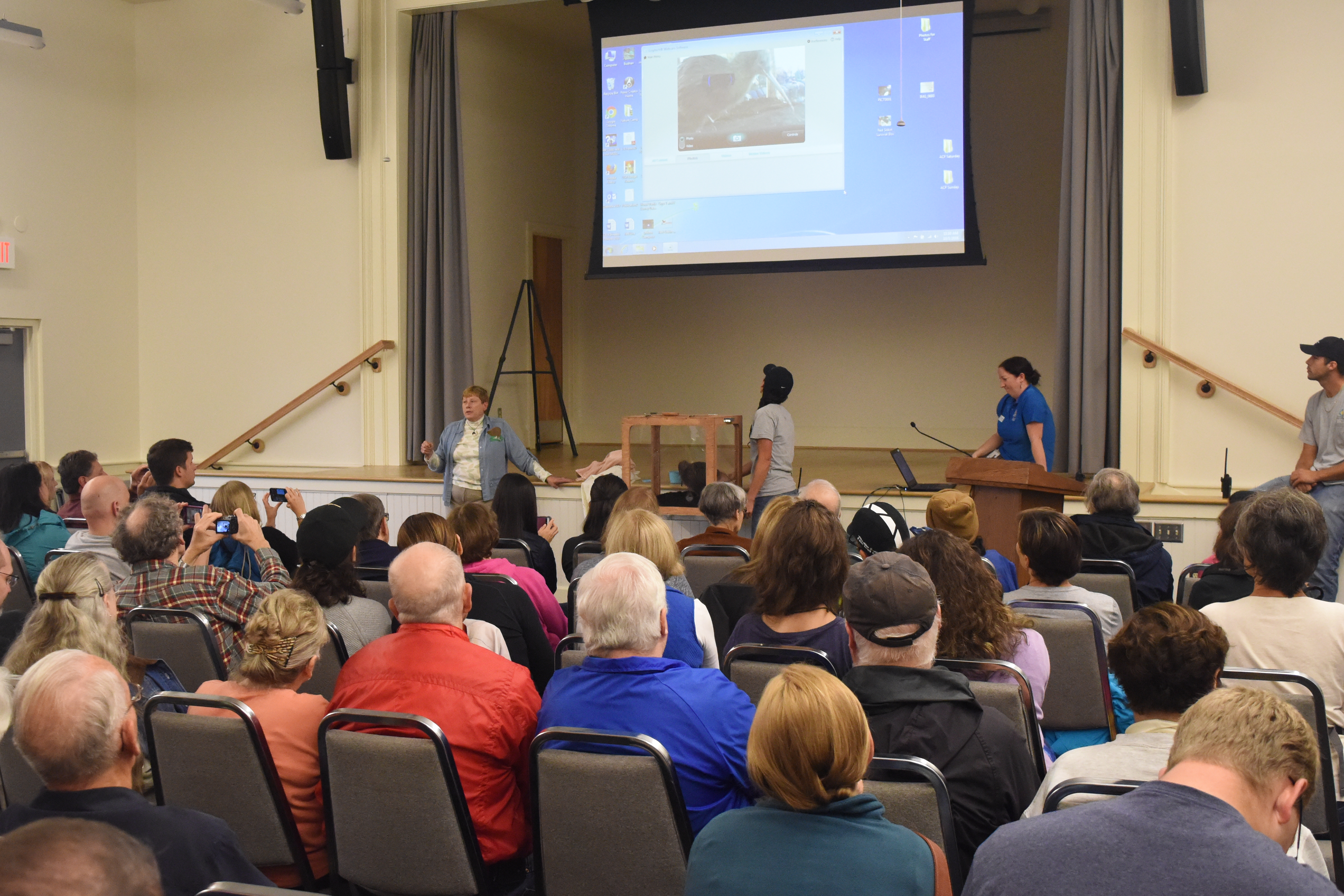

Supplement: Supplementary file 1 [file animals-15-01156-s001.zip › animals-3492583-supplementary/Supplementary Photo #4.JPG]
